# Fostering Quality Improvement Capacity in a Network of Primary Care Practices Affiliated With a Pediatric Accountable Care Organization

**DOI:** 10.1097/pq9.0000000000000175

**Published:** 2019-05-16

**Authors:** Charitha Gowda, Christina Toth, Suzanne Hoholik, Richard J. Brilli, Sean Gleeson, Stephen Cardamone

**Affiliations:** From the * Partners For Kids, Columbus, Ohio; † Nationwide Children’s Hospital, Columbus, Ohio; ‡ Division of Infectious Diseases, The Ohio State University College of Medicine, Columbus, Ohio; § Department of Quality and Patient Safety, The Ohio State University Wexner Medical Center, Columbus, Ohio; ¶ Division of Pediatric Critical Care Medicine, Department of Pediatrics, The Ohio State University Wexner Medical Center, Columbus, Ohio; ‖ Division of Ambulatory Medicine, The Ohio State University College of Medicine, Columbus, Ohio.

## Abstract

**Objectives:**

Quality improvement (QI) methodologies are not widely implemented in primary care practices. As an accountable care organization serving pediatric Medicaid recipients in Ohio, Partners For Kids (PFK) sought to build QI capacity in affiliated primary care practices to improve organizational performance on key quality measures.

**Methods:**

A team of QI specialists developed a comprehensive training program focused on pediatric QI initiatives. From 2014 to 2017, community-based, primary care practices affiliated with PFK were recruited to participate in QI. The primary outcome, assessed yearly, was the proportion of eligible PFK patients accessing care at a practice with ≥1 active QI project. The proportion of QI projects that demonstrated moderate improvement, defined as the implementation of ≥1 intervention and observed improvement in process measures, within 12 months of initiation was also calculated for 2017.

**Results:**

Over the study period, the PFK QI team supported 72 projects in 33 primary care practices throughout central and southeast Ohio. In 2017, 26 practices were engaged in ≥1 active QI project, reaching 26% of all eligible PFK patients. Of the 21 projects active as of January 2017, 11 (52%) showed moderate improvement within 12 months.

**Conclusions:**

The PFK QI team successfully supported QI capacity building in primary care practices throughout Ohio using a systematic approach to recruitment, training, and QI resource support. New, multilevel interventions are needed to promote the uptake of preventive services among patients.

## Abstract 2

Supplemental Digital Content is available in the text.

## INTRODUCTION

Rising U.S. healthcare costs, disproportionate to advances in quality, has led to alternative healthcare delivery and financing models. Specifically, accountable care organizations (ACOs) have emerged as a potential vehicle for delivering high-value health care, defined by the Triple Aim objectives—better individual care, improved population health, and lower per capita costs.^1,2^ Simultaneously, quality improvement (QI) has gained prominence as an effective methodology to marry the seemingly disparate goals of high-quality care and lower costs.

Although QI work has been pursued largely in hospitals or healthcare systems, primary care practices traditionally have not engaged in QI initiatives due to a lack of financial incentives and insufficient internal resources to support the work.^3^ However, within ACOs, primary care practices are recognized as central coordination centers to promote high-value care by focusing on preventive care and hospital avoidance. Also, payers increasingly are linking financial reimbursement to quality outcomes, potentially providing incentives for ambulatory practices to engage in QI.^4,5^

Partners For Kids (PFK) is an ACO composed primarily of community-based physician offices that have partnered with Nationwide Children’s Hospital (NCH; Columbus, OH) to oversee healthcare delivery for pediatric Medicaid recipients in Ohio. Over the past decade, NCH has developed a robust, internal QI infrastructure enabling it to reduce hospital harm events, mortality, and costs significantly.^6^ Recognizing the value potential for QI in the ACO, PFK and NCH leadership invested in building QI capacity in community primary care practices within the ACO network.

This report describes our experience engaging primary care practices affiliated with PFK in QI work. We chose 2 metrics to gauge the effects of QI within the practices: (1) the number of PFK patients potentially impacted by implemented QI projects, and (2) the level of QI engagement attained by the practices, as determined by the Institute for Healthcare Improvement (IHI) Collaborative Assessment Scale. We hypothesized that multiprovider group practices primarily serving Medicaid patients would be most likely to engage in QI.

## METHODS

### Study Population

PFK has medical and financial responsibility for 330,000 pediatric Medicaid recipients in 34 counties across central and southeast Ohio. As a Physician-Hospital Organization, PFK has direct contracts with over 1,400 providers in independent and NCH-employed practices,^7^ and its governance is shared equally between the hospital and representatives of affiliated physician groups. Although most PFK physicians are salaried employees of the hospital or practice partners, nonemployed community physicians receive Medicaid fee-for-service rates plus incentive payments.

Implementation of QI initiatives in primary care clinics has been shown to confer improved health outcomes for pediatric patients across disease states and over time.^8–11^ Thus, community primary care practices, providing care to ≥500 pediatric Medicaid recipients (n = 94), were considered eligible for participation in the PFK QI program. Approximately 228,844 children receive care in these practices and could potentially benefit from practice-based QI work. This work was deemed QI by the NCH’s Institutional Review Board and was exempt from further review.

### QI Program Development

In 2014, PFK built infrastructure for external QI practice facilitation by hiring one full-time QI specialist (QIS) who began by creating a portfolio of QI projects aimed at improving immunization rates, well-visit rates, asthma management, and antibiotic stewardship. A comprehensive QI training program was developed: three 1-hour interactive sessions designed to jumpstart participants on a QI project while instructing them on the QI methodology. Since 2014, the PFK QIS team has trained 173 individuals, including 80 community physicians and nurse practitioners and 93 office managers, medical assistants, and nurses. Participation was incentivized by offering Continuing Medical Education and Maintenance of Certification credit. To enhance the dissemination of best practices, we developed a quarterly, web-based seminar series. Didactic lectures on QI focus areas by pediatric experts were followed by interactive discussions among primary care practices to share project updates, challenges, and potential solutions.

### Practice Recruitment

**Fig. 1.**
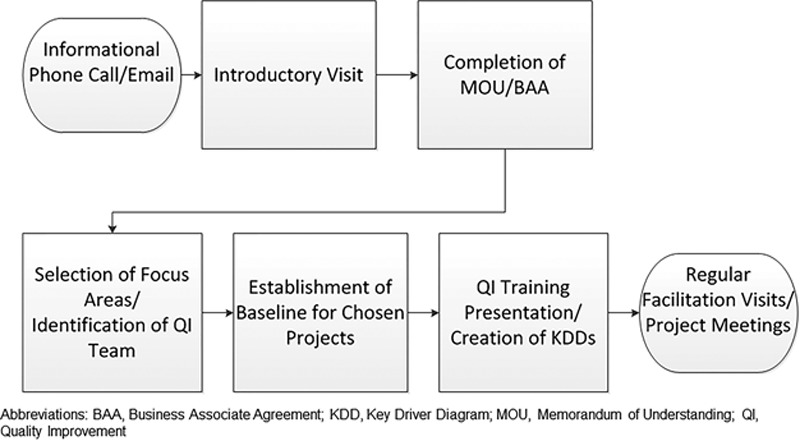
Process for engaging community primary care practices in the Partners For Kids’ QI Program. BAA indicates business associate agreement; MOU, memorandum of understanding.

Although practice facilitation for QI services was offered to all eligible practices, the QI team prioritized recruiting practices with historically poor performance on Medicaid quality measures and having large PFK patient volumes. A standardized process for engaging these community practices was followed (Fig. 1). Once a practice decided to participate, an internal QI team is formed consisting of a clinician lead and staff members. All team members underwent QI training, which included baseline measurement collection, project aim identification, and key driver diagram (KDD) development (see Appendix A.1, **Supplemental Digital Content 1**, http://links.lww.com/PQ9/A93). Practice teams typically selected projects based on identified patient needs and their baseline performance in relevant quality measures. The KDDs provided a clear overview of project outcome measures, baseline data, targets, and project timeline in the specific aim, and key drivers which should influence the outcome and interventions to help achieve defined goals.^12^ Although practice members were responsible for implementing interventions, the PFK QIS supported their efforts by providing data collection and analysis. Regular practice facilitation meetings were held to monitor progress and promote ongoing learning in QI methodology. By 2017, due to rapid program growth, the PFK QI team had expanded to 3 full-time QIS, all of whom had advanced training or prior work experience in QI.

### Study Outcomes

To assess the scope and efficacy of QI engagement across the pediatric ACO, we evaluated the proportion of patients accessing care at primary care practices engaged in QI and the level of QI engagement of the practices. Although we would have preferred to measure the proportion of patients involved in specific QI studies throughout the practices, substantial variation in data availability across practices prevented us from measuring that directly. We instead established the primary outcome as the proportion of eligible PFK patients who access care at a primary care practice that is actively engaged in QI and thus might benefit from the project(s). Attribution of PFK patients to a primary care practice was determined at the start of the calendar year and based on patients’ healthcare utilization over the prior 2 years. The primary outcome was calculated at the end of each calendar year.

To assess the level of engagement in each practice, we used IHI Collaborative Assessment Scale (see Appendix A.2, **Supplemental Digital Content 2**, http://links.lww.com/PQ9/A94). This validated metric assesses the level of engagement from “simply intending to participate” to “fully engaged with active projects attaining outstanding sustainable results.” Practices were considered actively engaged if they had participated in a QI project achieving a score of ≥1.5 on the IHI Collaborative Assessment Scale within the calendar year.^13^ Secondary outcomes included the proportion of practices with a QI project measuring ≥2.5 and ≥3.0 on the IHI Collaborative Scale within 6 and 12 months of initiation, respectively. To achieve scores of ≥2.5 and ≥3.0, respectively, practices had to start testing interventions and data collection and go on to demonstrate moderate improvement in process measures. These outcomes were intended to reflect practices that had clinically meaningful progress in their QI initiatives, similar to how the IHI Collaborative Scale has been used in other studies.^14,15^

### Data Collection and Analysis

The data repository of all healthcare claims submitted to PFK was accessed yearly to determine patient attribution. Quarterly, the QIS assessed each project’s performance using the IHI Collaborative Scale and identified practices with ≥1 active QI project during the calendar year. For each project, QIS maintained control charts for process and outcome measures to assess for statistically significant improvements over time.

Descriptive statistics of the QI program were completed annually from 2014 to 2017 on the relevant outcome measures (number of active QI projects and practices engaged in QI, and proportion of PFK patients seen at practices actively engaged in QI). For 2017, the number of QI projects measuring ≥2.5 and ≥3.0 on the IHI Collaborative Scale within 6 and 12 months, respectively, of their initiation were calculated. Specific characteristics of the engaged practices were described including practice setting, size, and patient volume. In unstructured interviews, the QIS team provided qualitative information to contextualize the program’s success in recruitment and project implementation.

## RESULTS

**Fig. 2.**
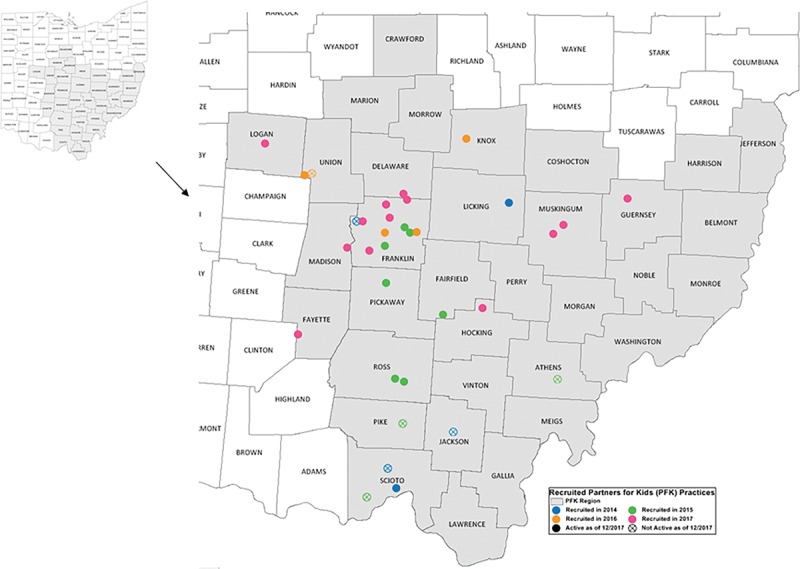
Map of Ohio showing the distribution of primary care practices that have engaged with the PFK Quality Improvement Program from 2014 to 2017.

Since 2014, the PFK QIS team supported QI capacity building in 33 community primary care practices located through Central and Southeast Ohio (Fig. 2). As of December 2017, 26 practices (79%) have maintained active projects, transitioning from requiring monthly to quarterly visits by PFK QIS. Seven practices (21%) no longer have active projects.

**Fig. 3.**
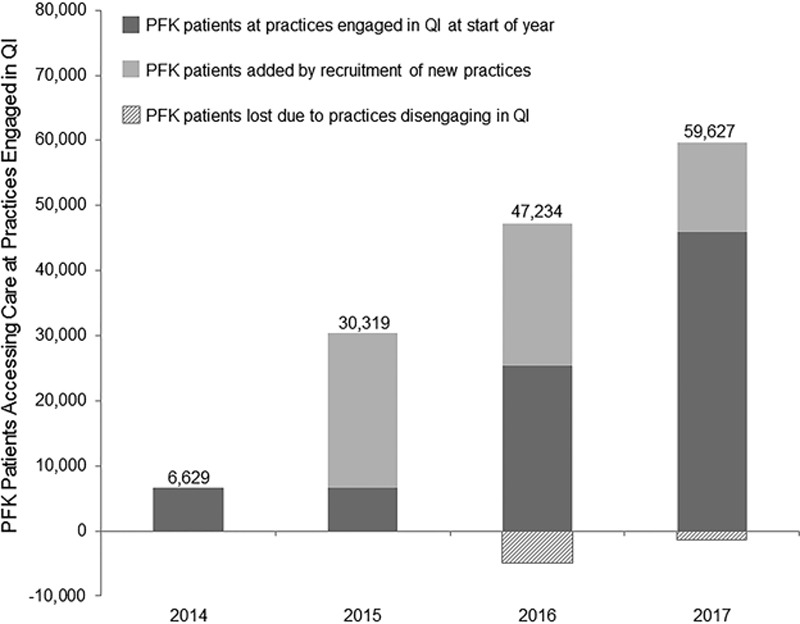
The growth of the PFK QI Program over time, as measured by *the number* of PFK patients who access care at a primary care practice engaged in QI.

The number of PFK patients who accessed care at a practice actively engaged in QI rose from 6,629 in 2014 to 59,627 in 2017, representing 26% of all eligible patients (Fig. 3).

During the study period, 72 QI projects were initiated and supervised. A median of 2 QI projects (interquartile range, 1–3) was carried out per practice, with a maximum of 6 projects in 1 practice. As of December 2017, 43 projects remain active.

There were 21 projects, implemented in 15 different practices, that have been active with long enough follow-up to allow assessment of engagement level by the IHI Collaborative Scale. Among these 21 active projects, 16 (76.2%) projects across 11 practices demonstrated measurable progress, having tested interventions and started data collection on key measures (≥2.5 on IHI Collaborative Scale), within 6 months of initiation. Furthermore, 11 (52.4%) projects demonstrated successful implementation of ≥1 intervention and showed moderate improvement in process measures (≥3.0 on IHI Collaborative Scale) within a year of project initiation.

The portfolio of QI projects has evolved in response to feedback by community practitioners and changes in Ohio’s Medicaid quality program. The most commonly selected projects were as follows: (1) fluoride varnish application for children younger than 6 years of age (n = 17); (2) well-child visit rates (n = 10); and (3) reduction in unnecessary emergency department visits (n = 8).

Specific characteristics of the community practices engaged in QI are presented in Table 1. The majority were multiprovider group practices serving ≥1,000 PFK patients and located in urban settings. Half of the practices reported prior QI experience, and 70% participated in multiple projects over the study period. Of the 23 practices engaged in multiple projects, most had multiple providers (n = 18; 78%), large staff (n = 19; 83%), and high patient volumes (n = 17; 74%).

**Table 1.**
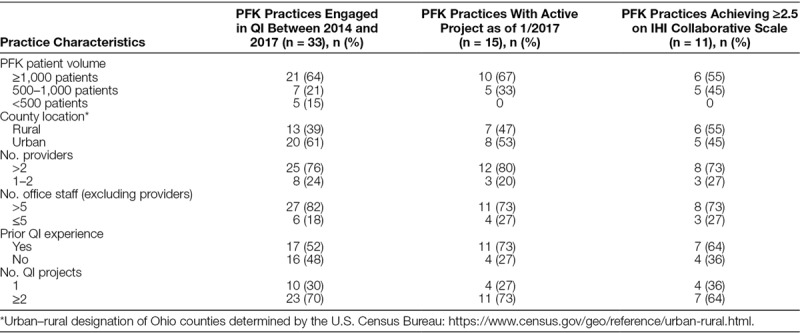
Characteristics of Practices at the Time of Initial Engagement With Partners For Kids (PFK) Quality Improvement (QI) Initiatives During the Study Period

## DISCUSSION

This study documents that, through external QI practice facilitation, primary care practices can successfully pursue QI initiatives individualized to the needs of their patients and/or clinical care staff, a process previously identified as essential to the concept of a patient-centered medical home.^3^ Further, although including actual results from individual studies is beyond this article’s scope, we have demonstrated that these projects can deliver significant QI as measured by high engagement scores on the IHI Collaborative Scale. We suggest that this effort’s success was based on elements related to *practice recruitment*, *project implementation*, and *ongoing support* of projects leading to improved outcomes.

### Practice Recruitment

We attribute our success in recruiting diverse practices in part to our emphasis on individualized approaches developed in conjunction with practice staff, frequent interactions between practice staff and QIS early on to build a working relationship, and opportunities for shared learning (eg, QI webinars). Although we had hypothesized that practices with a large staff, majority Medicaid patients, and prior QI exposure would be more likely to engage with us, no specific practice characteristics emerged that consistently predicted a practice’s response. We observed anecdotally that practices with QI experience were among those most resistant to engage, possibly due to prior negative associations such as excessive documentation requirements or limited external support to carry on projects. Participating practices ran the gamut from solo providers to multisite group practices with a corporate office. They were located in urban and rural areas and had considerable variation in payer mix among their patients.

### Project Implementation

The PFK QIS team did not adopt a “one-size fits all” approach to establishing QI projects within different practices. However, common themes emerged were effectively reused. For example, in practices that were concerned about having insufficient resources for QI work, the QIS team would recommend starting with a simple QI project that could be seen as an “easy win” opportunity, such as the promotion of fluoride varnish application during clinic visits. For that project, most interventions, such as training on fluoride varnish application, were directed primarily at the practice staff, instead of patients whose behaviors may not be as readily influenced (see Appendix A.1, **Supplemental Digital Content 1**, http://links.lww.com/PQ9/A93, for sample KDD). Further, interventions and change concepts could be quickly tested, allowing for a short timeframe between project initiation and outcome assessment. Therefore, practice members quickly acquired experience in QI methodology and came to view QI projects as feasible for their organization. Unlike prior QI experiences, practices felt supported throughout the QI process by our team’s practice facilitation. They then felt empowered to pursue more complex projects, although there was variation in the likelihood of and pace at which practices expanded their QI engagement. Practices with multiple providers and large ancillary staff were more likely, once engaged, to take on multiple projects, often implementing different projects at other practice locations.

### Ongoing Support

The key characteristics associated with long-term retention in QI projects were the assembly of an effective QI team including office staff, who typically implemented interventions, and the selection of a clear project lead within the practice.^16,17^ PFK community practices that effectively transitioned project leadership despite staffing turnover and expanded their QI team to include medical assistants and office receptionists often successfully pursued multiple QI projects over time. Ultimately, among small and large practices alike, the presence of an enthusiastic QI champion often was the deciding factor for a practice’s likelihood to have multiple projects running concurrently. On the other hand, the primary factors that led community practices to dissolve their QI partnership with PFK over the study period were lack of these elements.

### Ongoing Challenges

#### Physician/Staff Engagement

We tried many approaches to convince physicians and staff to participate in the PFK QI program. Proposing QI as a way to meet quality targets in an available incentive plan (wherein additional payments were provided if quality targets met) was effective for engaging some practices, as has been shown in other settings.^18,19^ However, we found that ongoing financial incentives were not necessary to retain practices in QI. After successfully participating in one project, practices appeared to add new projects based on the unique needs of their patients and regardless of whether the project measure was part of the Incentive Plan. To date, the PFK Incentive Plan has been developed independently of the QI program by a committee of PFK staff members with consultation from community providers, but the effectiveness of financial incentives as a recruitment tool suggests that we should pursue greater alignment of quality goals between the Incentive Plan and our program in the future.

It is worth mentioning that PFK manages physician and health plan enrollment through the loose messenger model approach rather than the more common single-signature approach used in most Clinically Integrated Networks (CINs), thereby allowing each practice to select its level of participation with each plan.^20,21^ Most physician-driven ACOs are built as CINs, which adhere to strict requirements dictated by the U.S. Department of Justice, including compliance with care guidelines and measurable performance improvements.^22^ Physicians that fail to participate in CIN-sponsored improvement programs put their membership at risk. In contrast, PFK relies on practices’ voluntary participation in the QI program.

#### Patient/Family Engagement

For most of our QI initiatives, the QI team has worked with practices to adopt interventions focused primarily on influencing the behavior of physicians and staff. These interventions have been relatively straightforward to implement and have achieved early gains in the specific process and/or outcome measures being tracked. However, for continued progress in many of the chosen QI projects, the QI team has started exploring interventions that directly influence patients’ behavior, which can be more challenging to implement successfully. Patient-specific factors, including lack of health literacy, perceived unimportance of preventative care, or transportation unavailability,^23^ may impede the ability to engage patients. Partnerships among inpatient, outpatient, and community settings have been shown to improve asthma-related health outcomes for Medicaid-insured patients.^11^ As an ACO, PFK capitalizes on its strong physician–hospital partnership to pursue similar coordinated QI work, thereby reaching those patients accessing only hospital-based care.

Other strategies aimed at directly influencing patient behaviors have focused on multilevel interventions, recognizing that numerous factors often drive patients’ actions.^24^ Margolis et al^25,26^ pursued interventions that targeted not only ambulatory practices but also families and community health organizations to improve the delivery of preventive services to children within that community. Going forward, PFK will explore linking practices with community resources such as local health departments. Tapping into these existing resources could help community practices expand the support available to patients who otherwise cannot consistently access primary care. Last, patients and their family members can provide critical perspectives on existing barriers to care, and their participation in QI projects should be explored. Depending on the diversity of patients and perspectives targeted, the ideal approach may range from pursuing semistructured interviews or focus groups to having patients serve on the QI team.

A limitation of this study is that our experience promoting QI across a pediatric ACO caring for Medicaid beneficiaries in Ohio may not be generalizable to other organizations in other states. However, because our ACO is a large, diverse organization affiliated with community primary care practices of varying size and practice models, we believe that many of the improvement and practice partnership lessons are transferable to other settings. Also, PFK’s atypical contractual structure created a significant barrier to practice engagement and retention that other organizations may not face. PFK’s voluntary participation approach demanded a more flexible and time-intensive approach to QI engagement than the more typical CINs where participation in a QI program could be mandated.

## CONCLUSIONS

We demonstrated how external QISs could be deployed effectively to support QI work across a large, diverse group of community practices serving pediatric patients. By moving out of the hospital setting into primary care, QI programs can reach more patients and promote the appropriate use of preventive care, potentially reducing downstream utilization of more costly healthcare. Through our learning network, we plan to develop new strategies that support healthcare providers’ engagement in QI across care domains and focus on changing patients’ behaviors directly. Further work, including qualitative studies, is needed to advance our ability to operationalize and sustain QI in the primary care setting effectively. Such an emphasis on prevention throughout childhood is a proactive strategy to ensure improved health outcomes at lower costs not only for our children today but also for our adults of tomorrow.

## ACKNOWLEDGMENTS

The authors acknowledge all of the community primary care practices that have participated in the Partners For Kids Quality Improvement Program for their enthusiasm and commitment to quality improvement. The authors thank Dr. Terrance Davis for his critical review of the manuscript.

## DISCLOSURE

Dr. Gowda, Ms. Toth, Ms. Hoholik, Dr. Gleeson, and Dr. Cardamone were paid employees of Partners For Kids during the study period. Dr. Brilli is a chief medical officer for Nationwide Children’s Hospital, the partnering hospital of Partners For Kids.

## Supplementary Material
