# Supplementary figures and images for: Fostering Quality Improvement Capacity in a Network of Primary Care Practices Affiliated With a Pediatric Accountable Care Organization
Source: Pediatr Qual Saf. 2019 May 16;4(3):e175. doi: 10.1097/pq9.0000000000000175 (PMC6594781; doi:10.1097/pq9.0000000000000175)

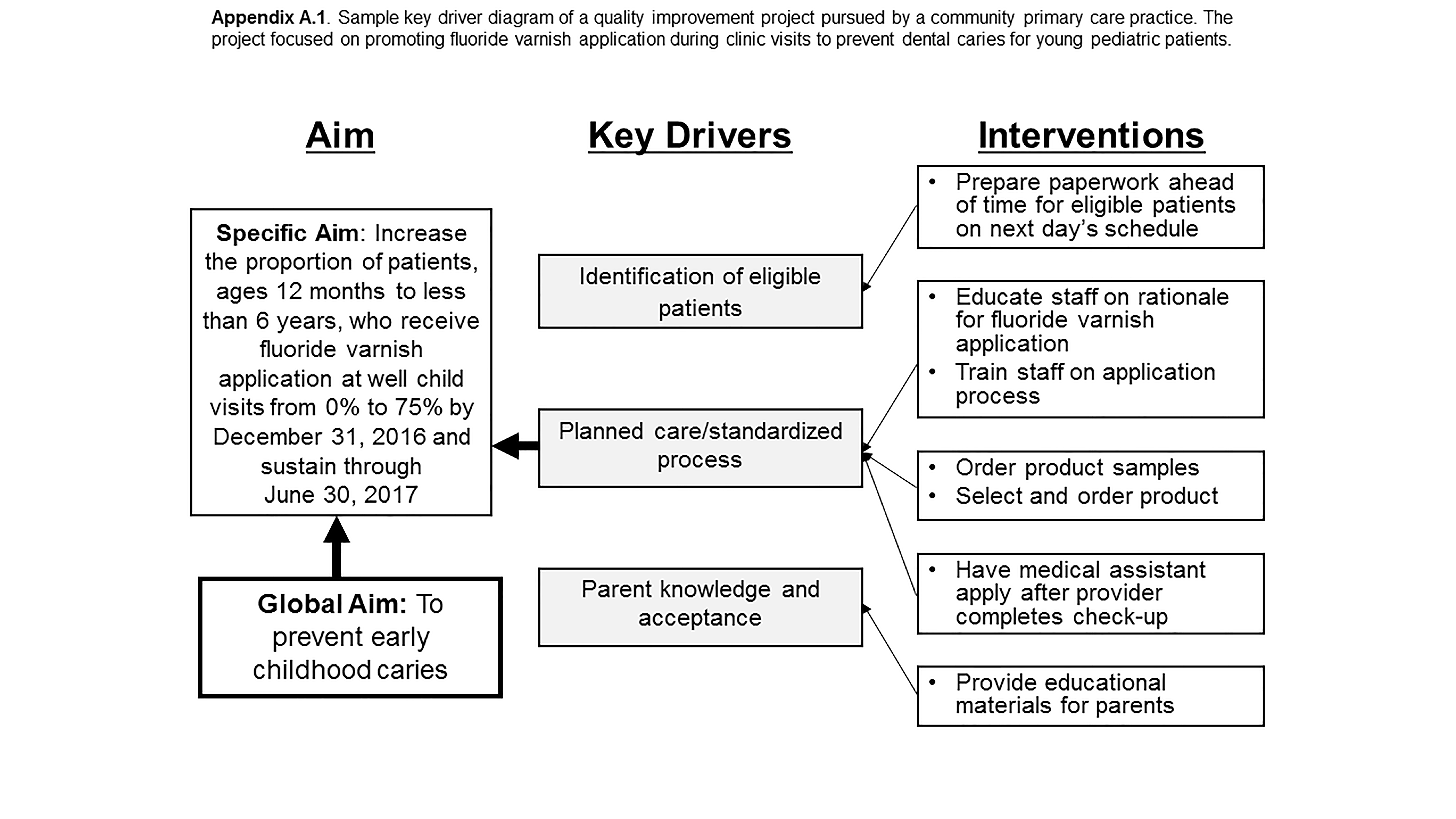

Supplement: Supplementary file 1 [file pqs-4-e175-s001.tif]

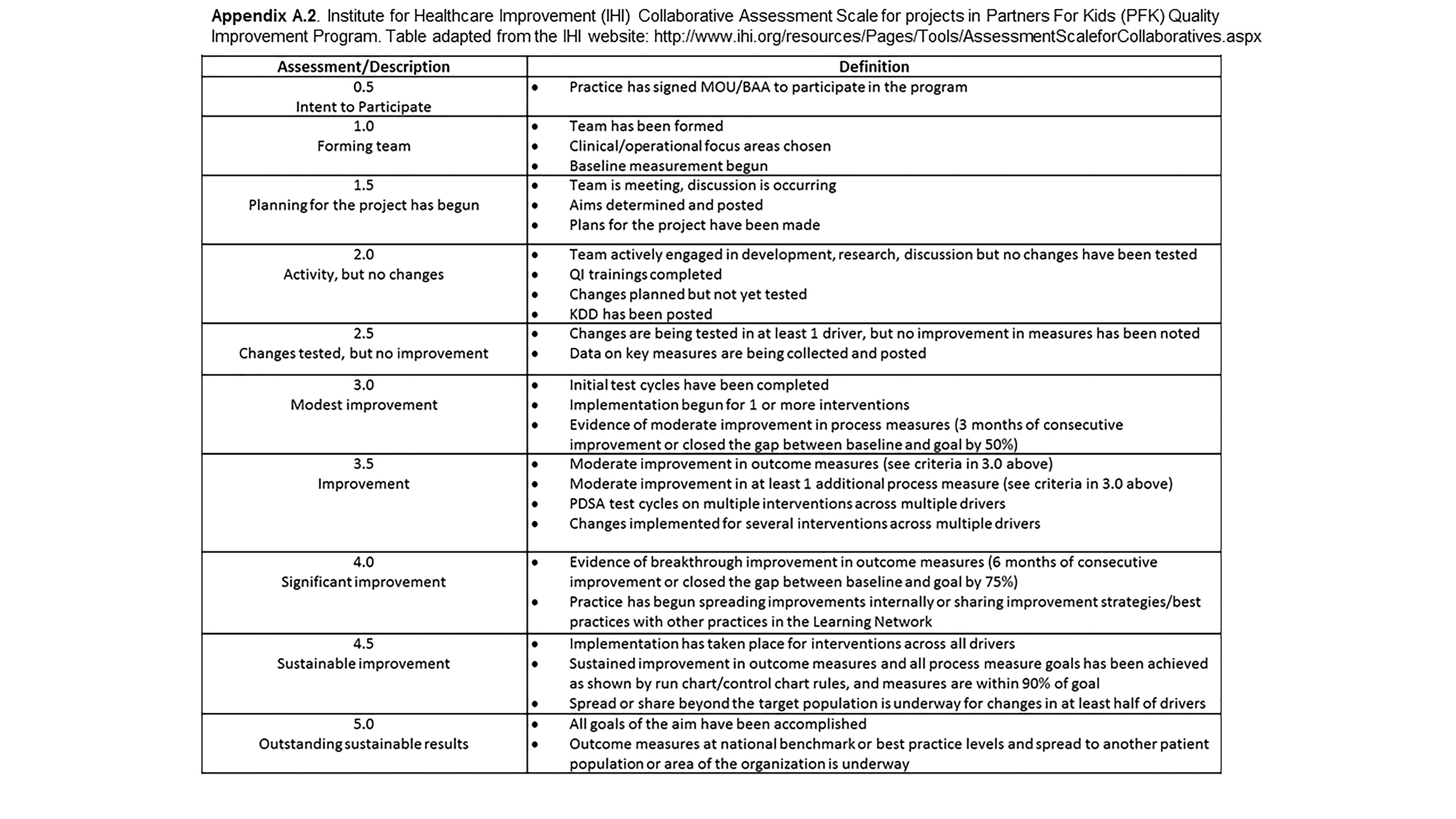

Supplement: Supplementary file 2 [file pqs-4-e175-s002.tif]
